# Connections Reduce Rheumatic Heart Disease‐Related Mortality in Western Australia: A Mixed Methods Study

**DOI:** 10.1111/ajr.70022

**Published:** 2025-03-10

**Authors:** Virginia DeCourcy, Daniel Hunt, Ingrid Stacey, Emma Haynes, Beverley Paterson, Clare Huppatz, Marisa Gilles, Judith Katzenellenbogen

**Affiliations:** ^1^ WA Country Health Service Perth Australia; ^2^ Derbarl Yerrigan Health Service Perth Australia; ^3^ Cardiovascular Epidemiology Research Centre University of Western Australia Perth Australia; ^4^ National Centre for Epidemiology and Population Health Australian National University Canberra Australia; ^5^ Department of Health Government of Western Australia Perth Australia

**Keywords:** Aboriginal and Torres Strait Islander health, cardiovascular disease, rheumatic heart disease

## Abstract

**Introduction:**

Preventable rheumatic heart disease (RHD) deaths continue to occur in Australia, with Aboriginal people disproportionately affected. Despite research into structural drivers and the lived experience of people with RHD, and national guidelines focusing on RHD prevention and treatment, recent coronial inquests have highlighted that systemic failures are ongoing. Few studies describe RHD service delivery and/or mortality within the Western Australian (WA) context.

**Objective:**

This study aimed to integrate quantitative information regarding RHD‐related deaths in WA between 2012 and 2021 with qualitative interview data to identify system‐level opportunities for the prevention of RHD‐related mortality in the WA health care setting.

**Design:**

Using quantitative data from the WA RHD register, a descriptive analysis of the clinical and demographic characteristics of RHD patients aged < 65 years was conducted, stratified by vital status. Thematic qualitative analysis of RHD stakeholder interviews was conducted in parallel, capturing systemic factors perceived to prevent or contribute to RHD‐related mortality in WA.

**Findings:**

Limited health service contacts were documented for the 60 registered‐recorded deaths among people with RHD. Interviewees emphasised that access to appropriate care was vital to prevent mortality. Passionate healthcare providers connect patients with care by fostering trusting relationships, but logistical, socio‐cultural and care quality barriers continue to hamper connections.

**Conclusion:**

Systemic change is needed in WA to support motivated providers and ensure that efforts to reduce RHD mortality do not rely on individual initiatives. This study contributes evidence for the need to improve RHD program design by prioritising patient‐provider connections, empowering, and resourcing providers to effectively engage with RHD patients.

**Summary**

- What is already known on this subject
  ○ RHD mortality rates, although declining, remain significantly higher in Aboriginal and Torres Strait Islander people in Australia compared to non‐Aboriginal people.
  ○ Aboriginal and Torres Strait Islander peoples face a range of complex barriers to accessing high‐quality healthcare, including for RHD treatment and management.
- What this paper adds
  ○ In Western Australia, RHD register data demonstrate limited health service contact among people who have died with RHD, including low utilisation of echocardiography, specialist, and secondary prophylaxis services.
  ○ The Western Australian health system is strengthened by motivated RHD health providers who are committed to building relationships with RHD patients to overcome access barriers and ensure access to best‐practice care.
  ○ Frontline providers can benefit from more support and training.
  ○ This study is the first mixed‐methods analysis investigating RHD mortality in Australia and the first to examine RHD‐related mortality in Western Australia.

## 1 Introduction

Rheumatic heart disease (RHD) is valvular heart damage that can develop after acute rheumatic fever (ARF), an immune reaction to a group A streptococcal infection. Australian rural and remote residents are disproportionately affected by RHD and face challenges accessing care compared to urban residents [1, 2]. RHD‐related mortality rates are declining globally; however, in Australia, preventable RHD deaths continue to occur and are particularly high among Aboriginal and Torres Strait Islander peoples (hereafter, respectfully, ‘Aboriginal’) [3, 4, 5]. Consequently, considerable Australian research in the last decade has investigated RHD acquisition and progression in Aboriginal communities [6, 7, 8] to reduce RHD‐associated morbidity and mortality.

Examination of the structural drivers of ill health has emerged as a priority for RHD prevention, notably as an alternative to deficit discourse, in which behaviours of Aboriginal people are blamed for poor health outcomes [9, 10]. Guiding documents, including the Australian ARF and RHD Guidelines [11] and the RHD Endgame Strategy [12], outline recommendations to avoid RHD harms. Central to these is the importance of health system improvements, including cultural safety, embedding environmental health in primary care, and building the Aboriginal health workforce. These recommendations stem from research into the lived experiences of people with RHD, who face significant barriers to accessing best‐practice RHD care [13, 14]. The impact of health system failures is highlighted in several recent publications and high‐profile coronial inquiries, including the RHD Doomadgee cluster in Queensland and the death of Mr. Yeeda in Western Australia (WA) [15, 16, 17].

Guideline‐recommended treatment for people with ARF or RHD is multifaceted, including ongoing frequent contact with primary and specialist healthcare providers [11]. Treatment includes regular secondary prophylaxis penicillin (BPG) injections, ongoing echocardiographic monitoring, and may also include surgery, medication, dental, and specialist pregnancy care. Jurisdictional RHD control programs have been established throughout Australia to support providers in better detecting, monitoring, and treating RHD and ARF. The WA RHD Register (hereafter ‘WA register’) was established in 2009 and is managed by the WA Country Health Service on behalf of the state [18].

Despite Australia's universal publicly funded health system and the above strategies, deaths from RHD continue to occur. The Queensland Coroner found that fractured relationships between health services, barriers to information sharing, and difficulties retaining skilled and culturally competent staff contributed to the RHD‐related deaths of three young Aboriginal women in 2019 and 2020 [15]. The WA Coroner found that communication challenges between health services and limited availability of culturally safe services contributed to missed opportunities for RHD treatment in the case of the 2018 death of Mr. Yeeda [16]. These coronial inquiries add to lived experience research findings that Australian health systems tend to provide individually focused RHD care that does not incorporate the worldviews of Aboriginal patients, many of whom are children and young people, or adequately involve their families and communities in decision‐making [13].

The narratives around these high‐profile deaths and qualitative research regarding system failures are reflected in quantitative studies reporting higher RHD mortality rates among Aboriginal people compared to non‐Aboriginal people, with substantial differences between 25 to 44‐year‐olds from these groups [4, 5]. However, few studies describe RHD mortality in WA specifically or examine WA's RHD service delivery context. Two WA studies examining data from the late 1990s to early 2000s found high RHD mortality among Aboriginal people but included Kimberley residents only [19, 20]. Other RHD mortality research aggregates mortality in WA with other jurisdictions and focuses on the Northern Territory where RHD prevalence is highest [4, 5, 21]. Between 2012 and 2022, age‐standardised RHD‐related mortality was 14.9 deaths per 100,000 person‐years for Aboriginal people aged < 65 in WA, a rate 38 times higher than that in non‐Aboriginal people [Table S1].

To date, quantitative studies focussed on RHD‐related mortality have not integrated qualitative data or theoretical perspectives such as Critical Race Theory or Standpoint Theory, narrowing contextual understanding of the statistics reported [22]. Therefore, the aim of the present study was to integrate quantitative WA Register information regarding RHD‐related deaths between 2012 and 2021 with qualitative interview data from RHD stakeholders to identify system‐level opportunities for the prevention of RHD‐related mortality in the WA health care setting. The specific objectives were to (i) describe clinical and demographic characteristics of RHD patients aged < 65 years from the WA Register, stratified by vital status and (ii) examine perspectives of practitioners working with RHD patients and their families regarding factors within the WA healthcare system that may contribute to or prevent RHD‐related mortality.

## 2 Methods

### 2.1 Author Position Statement

Virginia DeCourcy is a non‐Aboriginal woman who undertook this study during her Master of Philosophy in Applied Epidemiology in collaboration with one Aboriginal and six non‐Aboriginal co‐authors with substantial experience in Aboriginal health. The study was motivated by concerns about the contribution of health system issues to RHD outcomes for Aboriginal people in WA, emphasised by community and family members during team members' involvement with RHD deaths in clinical and coronial settings. An Aboriginal health advisory group comprised of three members with experience across government, clinical care, and research provided advice on scope, study design, and framing, and reviewed the final manuscript.

### 2.2 Objective 1 (Quantitative Methods)

#### 2.2.1 Data Source and Participant Selection

De‐identified person‐level records were sourced from the WA Register from 01/01/2012 to 31/12/2021 (extracted March 2023) for cross‐sectional analyses. The WA Register includes routinely collected variables associated with ARF and RHD diagnosis, treatment, and noviewees were invited to provide feedback on findtification. Date and cause of death information are also recorded within the WA Register upon notification from healthcare providers or after monthly review of hospital records.

De‐identified person‐level records were sourced from the WA Register from 01/01/2012 to 31/12/2021 (extracted March 2023) for cross‐sectional analyses. The WA Register includes routinely collected variables associated with ARF and RHD diagnosis, treatment, and notification. Date and cause of death information are also recorded within the WA Register upon notification from healthcare providers or after monthly review of hospital records.

Registrants with a RHD diagnosis on or before 31 December 2021 were included in the analysis, with stratification by vital status. Persons with a date of death recorded, who were aged < 65 years at the time of their death during the 2012–2021 study period, were allocated to the “deceased group”; persons aged < 65 years on 31/12/2021 without a recorded death were allocated to the “prevalent group”. Participation was restricted to < 65‐year‐olds to focus on premature mortality, maintain consistency with the earlier analysis of death registry [Data S1], and to allow comparison with similar Australian research [4].

#### 2.2.2 Variables Analysed

Demographic characteristics included year of birth, sex (male, female), ethnicity (Aboriginal, Aboriginal and Torres Strait Islander, Torres Strait Islander, Māori, Pasifika, Asian, African, Middle Eastern, other Australians), and region of primary health provider in WA (metropolitan, Kimberley, Pilbara, Midwest, Wheatbelt, Goldfields, South West, Great Southern). For analysis, values of ethnicity were allocated as either Aboriginal (including Torres Strait Islanders) or non‐Aboriginal, and regions outside the Kimberley and Pilbara were condensed into a single ‘other regional’ category due to low numbers.

Clinical details included dates of recorded ARF episodes and RHD diagnoses; dates of BPG doses administered; dates and medications for secondary prophylaxis prescriptions; dates and categories of recorded RHD‐related surgeries (repair, replacement, valvuloplasty); dates of the most recent echocardiogram and the most recent specialist review for RHD, and RHD severity recorded at the most recent echo (ARF only, mild, moderate, severe). ‘Time since’ variables were derived as the difference between treatment/service and study exit (death date or 31/12/21 as appropriate). The cause of death category (RHD‐related, non‐RHD, unknown) was derived from the free‐text cause of death variable recorded in the WA register and reviewed by a medical professional.

BPG adherence was estimated pragmatically for the 12 months before exit from the study for each person by dividing the number of BPG doses received by the number of doses expected [11]. Expected doses were estimated based on the fact and frequency of BPG prescription in the 12 months before study exit. Persons without a prescription for BPG prophylaxis were excluded from this calculation.

#### 2.2.3 Statistical Analysis

Frequencies and proportions were calculated for demographic and clinical covariates within the deceased and prevalent groups to assess patterns of distribution. Summary statistics (median and range) were calculated for continuous variables. Registrants with missing values were excluded from the analysis of relevant variables.

No formal statistical comparisons were made between the deceased and prevalent groups due to the descriptive aims of the quantitative analyses.

Analyses were conducted in SAS v9.4.

### 2.3 Objective 2 (Qualitative Methods)

#### 2.3.1 Study Design

The qualitative analysis of semi‐structured interviews sought to identify systemic issues contributing to RHD mortality in WA, rather than focusing on individual behaviour or decisions. Cognisant of the sensitivity of mortality discussions with families, the study focused on stakeholders who had been involved with RHD patients through their work in health or other government systems.

#### 2.3.2 Participant Selection

Stakeholders were identified using purposive and snowball sampling, were provided information about the study aims, and invited to participate via direct email. Sampling sought to identify stakeholders with a range of experience working with WA RHD patients and their families.

#### 2.3.3 Data Collection

An interview guide was developed in consultation with an Aboriginal health advisory group [Data S2]. One‐on‐one interviews were conducted via Microsoft Teams between December 2022 and May 2023 by Virginia DeCourcy, ranging from 40 to 75 min. Interviewees were asked open‐ended questions about their experience with RHD patients, their perspectives on the health system's role in RHD patient journeys, and what factors might be contributing to mortality. Further questioning was responsive to topics raised by interviewees. Interviews were recorded, transcribed, and checked by participants before analysis. Notes were made following each interview and discussed with the research team along with interview techniques and emerging themes.

#### 2.3.4 Data Analysis

Thematic coding of transcripts was conducted in NVivo by Virginia DeCourcy and initiated from midway through data collection. Themes were generated inductively from interview data. In consultation with co‐authors, a codebook was developed and updated iteratively to incorporate additional data. Data analysis followed a theory‐building approach and was informed by insights into the correlation between RHD patient experience, potential causes of mortality, and systemic issues. Interviewees were invited to provide feedback on findings.

## 3 Results

### 3.1 Quantitative Analysis

The initial data extract included 1281 registrants listed on the WA Register on 31/12/21. After exclusion of registrants without a recorded RHD diagnosis, those who died before the study period, and those aged > 64 at study exit, 820 registrants were included in the analysis. Of these, 60 (7.3%) had a recorded death between 2012 and 2021 (deceased group), and 760 were alive on 31 December 2021 (prevalent group) [Table 1].

**TABLE 1:** Characteristics of RHD patients aged < 65, WA register, 2012–2021.

Characteristic	Deceased group (%)	Prevalent group (%)
TOTAL	*N* = 60	*N* = 760
Age at study exit		
Median	45.5	31.0
Age group		
0–14	0 (0%)	79 (10%)
15–24	< 5	185 (24%)
25–34	9 (15%)	164 (22%)
35–44	13 (22%)	153 (20%)
45–54	22 (37%)	115 (15%)
55–64	12 (20%)	64 (8%)
Ethnicity		
Aboriginal and/or Torres‐Strait Islander	59 (98%)	700 (92%)
Sex		
Female	32 (53%)	510 (67%)
Region		
Kimberley	40 (67%)	420 (56%)
Pilbara	9 (15%)	89 (12%)
Other regional	5 (8%)	134 (18%)
Metropolitan	6 (10%)	110 (15%)
Diagnoses		
Median age at RHD^a^ diagnosis	31.5	20
Persons with recorded ARF^b^ diagnosis	24 (40%)	498 (66%)
Median age at first ARF diagnosis	13	12
BPG^c^ prophylaxis in 12 months before study exit		
Persons with prescription	14 (23%)	498 (66%)
Median BPG adherence %	31	46
BPG adherence 80% or higher	< 5	90 (18%)
Previous surgery for RHD		
Persons with history of surgery	28 (47%)	151 (20%)
Count of procedures	47	245
Valve replacement	36 (77%)	153 (62%)
Valve repair	7 (15%)	65 (27%)
Valvuloplasty	< 5	27 (11%)
Echocardiography		
Persons with any echo recorded	60 (100%)	760 (100%)
Time since most recent echo		
Median in days	556	480
< 6 months	11 (18%)	169 (22%)
6 months–< 1 year	9 (15%)	143 (19%)
1 year–< 2 years	18 (30%)	171 (23%)
> 2 years	22 (37%)	277 (37%)
RHD severity at most recent echo		
ARF only	< 5	90 (12%)
Mild	8 (14%)	247 (33%)
Moderate	7 (12%)	191 (25%)
Severe	43 (73%)	229 (30%)
Missing	< 5	< 5 (< 1%)
Specialist review		
Persons with specialist review recorded	54 (90%)	715 (94%)
Time since most recent specialist review		
Median in days	602.5	588
< 6 months	11 (20%)	146 (20%)
6 months–< 1 year	10 (19%)	110 (15%)
1 year–< 2 years	15 (28%)	147 (21%)
> 2 years	18 (33%)	312 (44%)
TOTAL	60 (100%)	760 (100%)

^a^ Rheumatic heart disease.

^b^ Acute rheumatic fever.

^c^ Benzathine penicillin G.

### 3.2 Data Completeness

Data completeness for age, ethnicity, and sex was 100% for both groups. There were seven missing values for the region of primary healthcare provider among the prevalent group.

### 3.3 Characteristics of Prevalent Group

Among the prevalent group, the median age at study exit was 31 years, two‐thirds were female, and 92% were Aboriginal. Two‐thirds (68%) had their primary health care provider in the Kimberley or Pilbara, whereas 33% were in metropolitan or other regions.

Median age at RHD diagnosis among the prevalent group was 20 years, with 66% also having a recorded ARF diagnosis. One‐fifth had a recorded surgery, with proportionally more repair or valvuloplasty procedures (38%) than in the deceased group. Among the 30% of the prevalent group with severe RHD, 31% had an echocardiogram (median time since echo 480 days) and 25% had seen a specialist in the 6 months before study exit.

### 3.4 Characteristics of Deceased Group

Among the deceased group, the median age at death was 45.5 (ranging from 15 to 64 years), with four deaths in under 25‐year‐olds [Table 1]. Most were Aboriginal (98%) and half were female. Most (82%) had their primary healthcare provider in the Kimberley or Pilbara. The median age at RHD diagnosis was 31.5 years, and only 40% had a recorded ARF diagnosis. Almost half of the group (*n* = 28, 47%) had received surgery—a total of 47 RHD surgical procedures were performed, with over three quarters of these being valve replacements.

**FIGURE 1:**
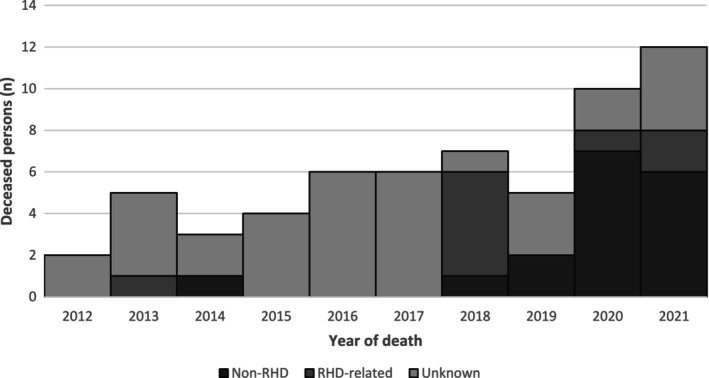
Mortality among RHD patients < 65, WA register, 2012–2021, by year and cause of death.

The number of deaths increased across the ten‐year period, from two deaths in 2012 to 12 deaths in 2021 (Figure 1). Overall, 57% of deaths had no known cause recorded in the register, while 15% were RHD‐related and 28% were not directly related to RHD. The recorded cause of death increased over the study period.

Severe RHD at the most recent echocardiogram was recorded for 73% of the deceased group, and the median time since the most recent echocardiogram was 556 days. Only 23% of those with severe RHD had an echocardiogram within six months before study exit, the guideline for patients in this category [11]. One quarter of those with severe RHD had seen a specialist in the six months before their death.

**FIGURE 2:**
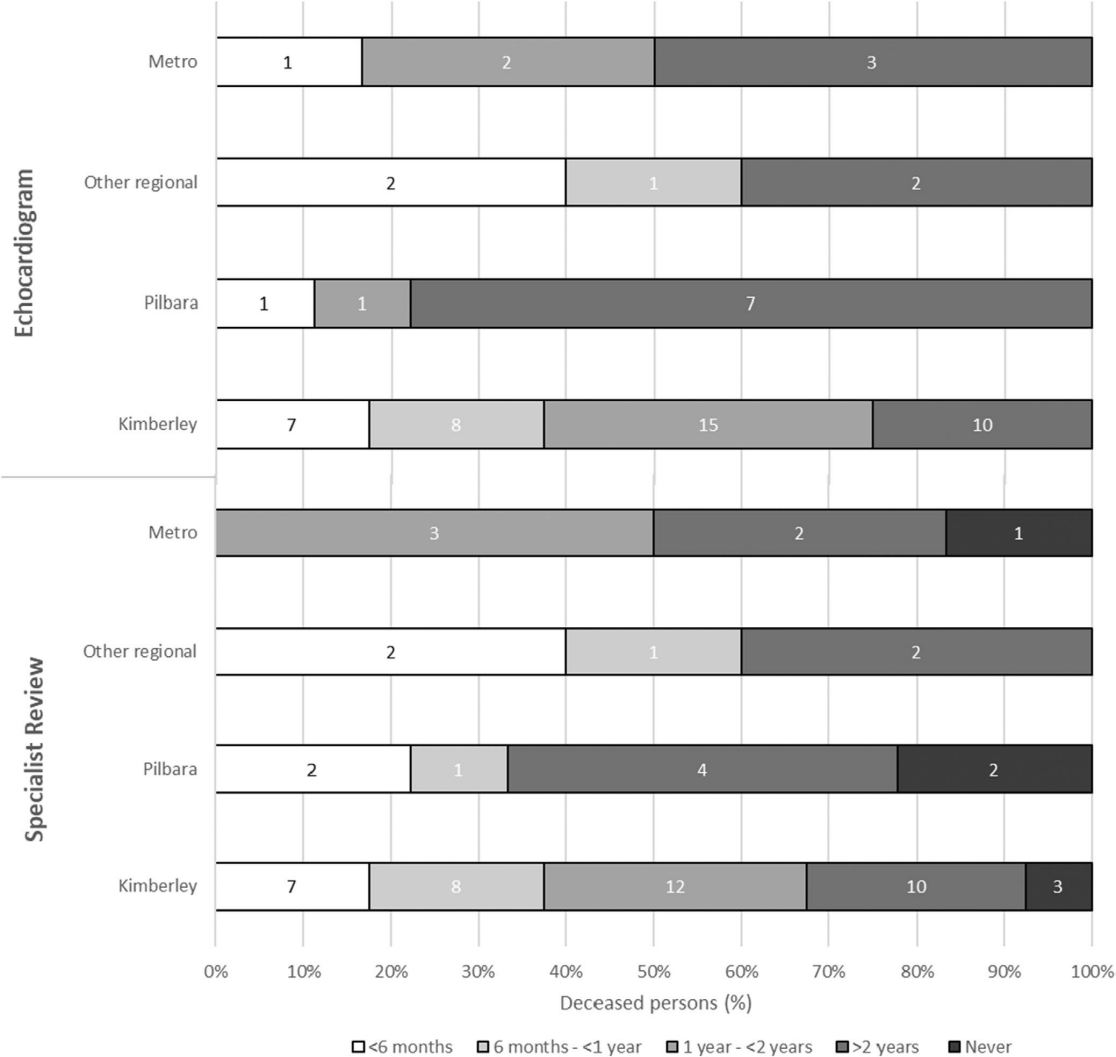
Time since health contacts among deceased RHD patients < 65 on the WA Register, 2012–2021, by region.

Noting small counts, Figure 2 suggests regional differences in the time since health contacts among the deceased group. For echocardiograms and specialist review, RHD patients in ‘other regional’ areas had the shortest time since the most recent contact, with over 50% seen within one year. Conversely, RHD patients in the Pilbara had the longest time since health contacts, with seven of nine people having more than two years since their most recent echocardiogram and six having more than two years since their most recent specialist review, or none recorded at all. Among Kimberley cases who died, 75% and 68% received echocardiograms or specialist reviews within the two years prior, respectively.

### 3.5 Qualitative Analysis

Of 24 stakeholders approached to participate, nine consented to an interview. Details of interviewees are outlined in Table 2. Seven interviewees were based in WA, and two were based in other jurisdictions but had experience working with WA RHD patients.

**TABLE 2:** Characteristics of interview participants, 2022–2023.

Characteristic	Participants (*n*)
Total	9
Women	6
Men	3
Aboriginal	3
Non‐Aboriginal	6
Experience^a^	
Primary care	6
Specialist care	2
Care coordination	1
Patient advocacy	2
Research	3

^a^ Participants with experience across multiple sectors are included in more than one category.

**FIGURE 3:**
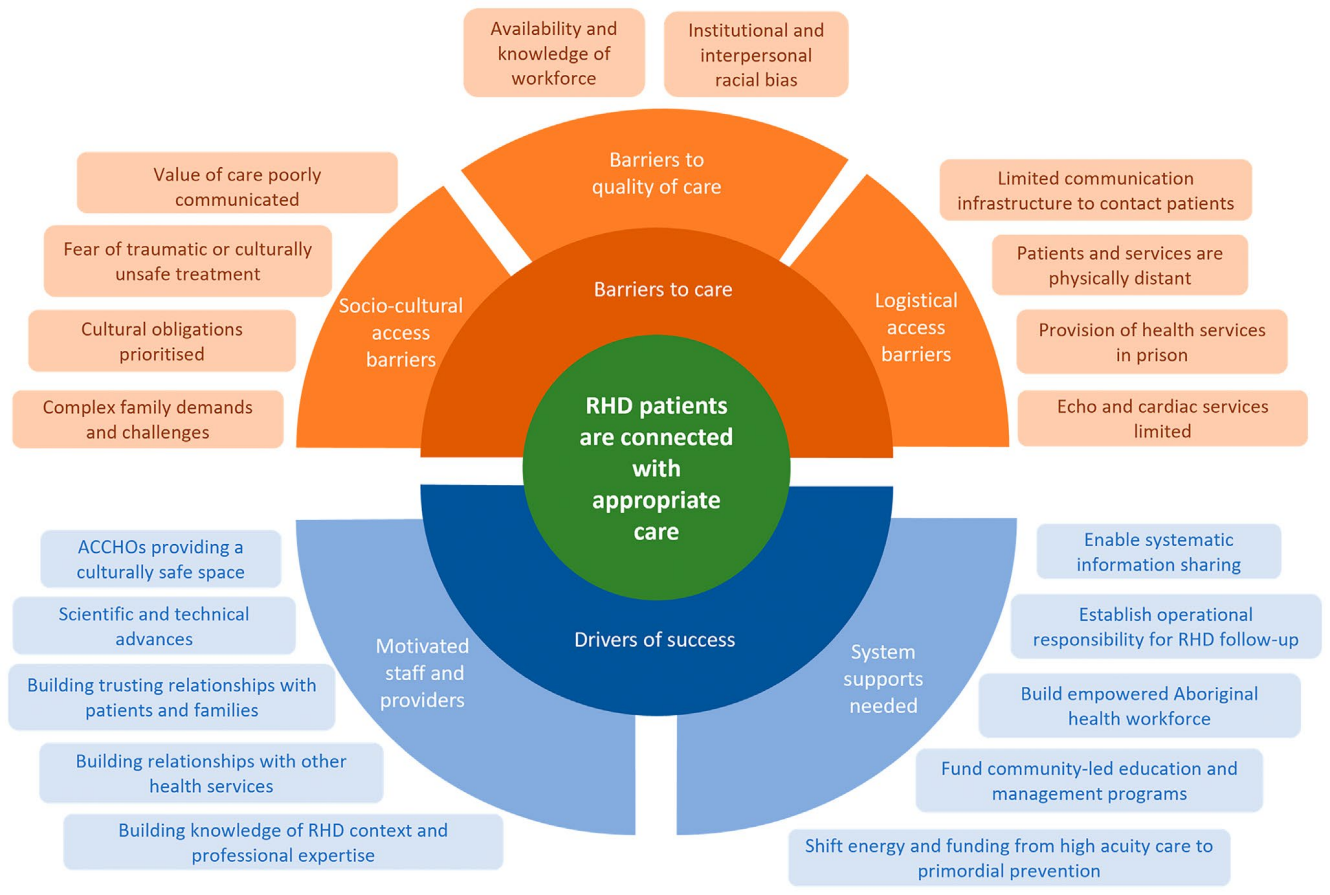
Theory of centrality of connections in prevention of RHD mortality generated through qualitative analysis.

Themes emergent in the interview data focus on the delivery of appropriate care to RHD patients [Figure 3]. Interviewees explained that missing secondary prophylaxis, echocardiography, or specialist review could result in unmonitored disease progression, and that access to surgical intervention and holistic follow‐up care was vital for patients with severe RHD. Interviewees stressed the importance of rapid intervention for acutely unwell RHD patients. The barriers to care and drivers of appropriate care are described in detail below. Aboriginal status is identified alongside interviewee quotes below, but no further details are provided to maintain confidentiality.

#### 3.5.1 Logistical Access Barriers

Interviewees described how health services in WA face practical constraints in reaching RHD patients.

Physical distance was a frequently cited barrier, as RHD patients residing in rural and remote areas face long and daunting journeys to travel to health services, often complicated by transport availability, additional expenses, and weather. This difficulty was highlighted by one Aboriginal interviewee who spoke about investing significant resources to get a patient to the airport, only for them to be turned away because of incorrect footwear. However, another interviewee emphasised that physical proximity alone could not facilitate access and that “some of the most disengaged clients we have live two minutes from this hospital”*[non‐Aboriginal]*.

Interviewees spoke about the importance of mobile cardiology clinics, which provide semi‐regular specialist services and echocardiography to rural areas across WA, improving access for remote patients who would otherwise need to travel to Broome or Perth for these services. However, interviewees also discussed the constraints of visiting services, including their limited frequency and locations. One interviewee noted that visiting clinicians tended to “sit in the consulting room and expect people to come”*[non‐Aboriginal]*, which was not sufficient to ensure access. Several interviewees noted that the establishment of permanent cardiology services across rural WA would be preferable but perceived that this was unfeasible.

Other logistical barriers mentioned included the difficulty of contacting patients who may not have a fixed address, phone number, or internet access to organise appointments; and the challenge of providing comprehensive care to RHD patients in prison due to limited integration between health and justice medical systems.

#### 3.5.2 Socio‐Cultural Access Barriers

Interviewees outlined a range of socio‐cultural barriers that RHD patients face when accessing health services, even if logistical challenges can be overcome, with Aboriginal RHD patients in particular facing complex barriers.

Several interviewees remarked that Aboriginal knowledge systems do not align with a biomedical framework. Where health providers do not have the cultural understanding or time to explain the need for ongoing RHD treatment in a meaningful way for Aboriginal patients, the value of RHD care may not be fully understood and seeking out care is not prioritised. This is exacerbated when patients are not experiencing RHD symptoms, or “if you're a young, otherwise fit person who doesn't think of yourself as someone with a chronic illness”*[non‐Aboriginal]*. Interviewees noted that this barrier was especially prevalent among adolescents.

The importance of meeting cultural obligations and managing other complex family demands were also discussed as barriers to RHD care for Aboriginal patients/families/carers. Interviewees noted that RHD appointments were “taking people away from their own life”*[non‐Aboriginal]*, and that sorry business (funeral/bereavement obligations) and caring responsibilities often take precedence. Interviewees noted that Aboriginal RHD patients “live in varying degrees of complex life and dysfunction”*[Aboriginal]*, and may be experiencing family violence, poverty, difficulty with school engagement, and other non‐RHD health conditions simultaneously, making healthcare access challenging.

Interviewees spoke about how fear of a poor or culturally unsafe health care experience was a barrier to care. The pain associated with BPG injections can make patients reluctant to return, especially young children and their families. Surgical procedures in unfamiliar hospitals can be particularly daunting, especially where patients “believe that hospitals are a place to go to die, due to generational trauma”*[non‐Aboriginal]*. However, routine appointments can also be traumatic if providers are not perceived to be respectful, kind and empathetic. One interviewee commented that “some nurses feel that…they're a bit better than an Aboriginal person and they growl at them, and talk to them like a child…and that makes fear”*[Aboriginal]*.

#### 3.5.3 Barriers to Quality of Care

Even when RHD patients overcome barriers to access a service, the care provided may be regarded as inadequate. All nine interviewees mentioned transient workforce impacts, with new or shorter‐term staff frequently struggling to recognise and understand RHD, which is rarely seen in metropolitan settings. This results in missed opportunities for diagnosis and ongoing management, because “they just might not be aware of how detrimental [RHD] can be to somebody if they're left unmonitored”*[Aboriginal]*. Several interviewees also spoke about how racial bias could affect care, including Aboriginal patients being stereotyped as drug‐seeking or alcohol‐affected when they attend hospital; treatment being refused even after repeat visits; or health providers “giving up on our mob without understanding the reasons behind why they may be not showing up to an appointment”*[Aboriginal]*.

#### 3.5.4 Motivated Staff and Providers

While barriers are significant, interviewees spoke about how motivated staff “with a massive care factor”*[Aboriginal]* are working actively to overcome challenges and connect RHD patients with appropriate treatment. Building trusting relationships with RHD patients and their families was perceived as vital by interviewees, who described this as involving patients in shared decision‐making about treatments; providing outreach “embedded in the community as opposed to in a health setting”*[non‐Aboriginal]*; and being “emotionally motivated”*[non‐Aboriginal]* to improve patient outcomes. The strength of Aboriginal community‐controlled services in providing culturally safe care and maintaining strong relationships with RHD patients was highlighted.

Building strong relationships with other health services was also discussed as an important factor facilitating access to care, with interviewees describing how these relationships allowed for vital cooperation between primary and community health, hospitals, visiting clinics, and specialists.

Health practitioners described taking steps to improve their knowledge and professional expertise in RHD to improve care delivery. Interviewees with experience in primary care spoke about training in specialised techniques for BPG injections; learning about their communities to inform their approach to care; and discussed the importance of current initiatives to implement non‐expert echocardiography screening services [23]. Scientific and treatment delivery advances such as a Strep A vaccine in development, virtual reality headsets to distract patients from the pain of an injection, and new technologies to improve BPG administration were raised as “game changers”*[non‐Aboriginal]* with the potential to improve patient outcomes.

#### 3.5.5 System Supports Needed

While the motivation, commitment, and continued energy of health staff were perceived to reduce barriers, interviewees emphasised that systemic changes are needed to ensure the sustainable provision of high‐quality care.

Interviewees observed that the health system is adept at providing high‐acuity care to seriously unwell RHD patients, including heart surgeries and emergency transport. However, they perceived that insufficient funding was directed to earlier intervention; “there is a huge focus on worst case scenario as opposed to that primordial prevention in our health system”*[non‐Aboriginal]*. Interviewees consistently explained that poor housing drives RHD progression, and expressed frustration that this was not being adequately addressed; “Our government should be ashamed of themselves, thinking it's ok for people to live like that”*[Aboriginal]*. Surgical treatments were seen as futile in this context: “it seems stupid that you're repairing their valves and then putting them back in that environment that causes acute rheumatic fever”*[Aboriginal]*. Increased funding for environmental health programs was suggested by multiple interviewees as a priority for reducing poor RHD outcomes.

Several interviewees emphasised that greater empowerment of the Aboriginal health workforce in WA would improve RHD outcomes. This included increasing the scope and responsibilities of Aboriginal Liaison Officers and Aboriginal Health Workers, who “often feel like they're just transport officers”*[non‐Aboriginal]* despite wanting to have a greater role in decision‐making and care provision. One interviewee stressed that “unless they're empowered…unless people listen to them, their role is just decoration”*[non‐Aboriginal]*.

Enabling more systematic information sharing, and establishing operational responsibility for follow‐up, were also suggested as vital for strengthening the health system's ability to track and treat RHD patients. Primary health staff spoke about the fragility of current systems, which largely rely on individual staff member initiative. They felt personally responsible for patient follow‐up and established their own methods to do this, because “there's no real recall system…no‐one is seeking [patients] out”*[non‐Aboriginal]*. Information from the WA Register was perceived as useful, but difficult and time‐intensive to access. Automated reminders, improvements to regional RHD coordination, and “a nurse that is just allocated to RHD alone”*[Aboriginal]* in high‐burden locations were suggested to improve patient follow‐up.

Interviewees noted that funding for RHD education and management programs that are community‐led, and “embedded with community and cultural meaning”*[Aboriginal]* was essential to improve RHD outcomes and connect patients with care.

## 4 Discussion

Aligned with a Critical Race Theory perspective, evidence generated by this study demonstrates that weak connections between patients and health systems negatively impact best‐practice healthcare, likely contributing to RHD‐related mortality in WA. RHD mortality among WA Aboriginal people [Tables S1–S3] is similar to that previously reported in Australia, with high premature mortality compared to the non‐Aboriginal population, even among young people [4, 5, 19, 20, 24]. Analysis of the WA register for 2012–2021 revealed 60 deaths among under 65‐year‐olds, 59 of whom were Aboriginal. Each of these deaths represents a person who died prematurely from a preventable disease, with profound impacts for their families and communities. Interviewees expressed that healthcare access barriers permeate the RHD landscape in WA, making it challenging for RHD patients to receive best‐practice care. The story of Mr. Yeeda, who died from RHD at 19 years old, places these mortality statistics and healthcare delivery experiences in context and draws attention to the need for systemic change in WA [16].

Limited health service contacts among the deceased group were observed in the WA register analysis; this is supported by the qualitative findings regarding weak connections between health services and patients. This is consistent with previously reported associations between experiencing RHD‐related complications and lack of recorded ARF in young Australians [24]. Rather than contradicting the well‐evidenced link between ARF and RHD aetiology, this relationship is indicative of a population for whom opportunities for early intervention, including diagnosis and treatment of Strep A infections, ARF diagnosis, RHD monitoring, and secondary prophylaxis, have been limited or missed entirely. Barriers to accessing early intervention and healthcare are heightened for Aboriginal people with RHD, for whom practical challenges are exacerbated by ongoing impacts of colonisation, including fear and mistrust of a biomedical system that often provides care in a manner inconsistent with their values, knowledge systems and priorities [13, 14, 17]. Australian research has reported, for example, fear of child removal as a strong motivator against seeking care among Aboriginal people [13, 25]. As a condition that often affects children and for which families can feel unfairly blamed, access to RHD care may be particularly impacted by this. In this context, the value of the Aboriginal community‐controlled health sector and RHD programs that are governed by and designed for Aboriginal communities is clear.

A range of intersecting challenges were identified in the qualitative analysis as potential contributors to the weak connections between patients and appropriate care, including geographical distance from health services, competing demands, and transient healthcare staff. The results demonstrate that barriers to RHD care can be experienced differently by patients based on contextual factors including age, sex, and location. Difficult access to RHD care among teenagers has been previously reported, as they struggle to prioritise care in the context of adolescence and may be lost in the transition to adult services [12, 13, 14, 26]. This was reflected in interviewee perspectives that teenagers can be reluctant to seek care and in the nine deaths observed among people aged under 34 in the quantitative analysis. While women experience higher rates of RHD than men and higher RHD mortality overall [5, 20, 21], some interviewees suggested that women were better engaged with RHD services, perhaps due to routine health contacts during pregnancy or stronger health‐seeking behaviour, which is possibly reflected in the lower proportion of females in the deceased group (53%) than the prevalent group (67%). Australian RHD guidelines advise that remoteness complicates service access and contributes to poorer outcomes [11], consistent with the quantitative results in which 82% of those who died were from the Kimberley and Pilbara regions. Particularly poor health access among the deceased group from the Pilbara mirrored interviewee comments about the region's restricted availability of echocardiography and specialist services. These findings highlight the need for age, gender, and location‐specific strategies to encourage access to RHD care.

Importantly, this study finds that even in the presence of significant barriers, stakeholders perceive that RHD care access can be facilitated by passionate healthcare professionals who understand their local communities. These dedicated staff are a precious resource within the WA system, likely contributing to the characteristics of the prevalent group, of whom 56% had accessed a specialist within the previous two years, despite barriers. Interviewees described being proactive in providing care to RHD patients, taking the initiative to deliver culturally relevant and age‐appropriate health messages, and feeling personally responsible for ensuring that patients accessed necessary care. Systemic changes, such as those outlined below, are needed in WA to support and complement these individually motivated actions and reduce RHD mortality.

Health providers who are Aboriginal play a vital role in overcoming care barriers by building therapeutic relationships based on cultural understanding and community knowledge [25, 27]. Additionally, while trusting relationships between non‐Aboriginal health providers, patients, and their families take considerable time and effort to develop, these are key facilitators of access to care [28]. Both Aboriginal and non‐Aboriginal interviewees emphasised the centrality of mutual respect and strong relationships to engaging with RHD patients, mirroring findings from earlier research with RHD practitioners [17]. Healthcare providers' acknowledgement of the cultural strengths of their communities, holistic approach to health and incorporation of this into healthcare provision is a key protective factor mitigating the impact of RHD in Aboriginal communities [14]. Resourcing, ongoing training and empowerment of the Aboriginal health workforce should therefore be prioritised to ensure sustainable access to culturally safe care for WA RHD patients, as outlined in existing recommendations [12, 17, 29]. For non‐Aboriginal healthcare providers, meaningful and context‐specific cultural safety training that prompts critical self‐reflection is needed, and has effectively shifted health professionals' attitudes and behaviours in the Northern Territory [30].

Exposure to poor RHD outcomes takes a toll on health providers, with feelings of hopelessness, frustration, and burnout resulting from systemic failures [9]. In this study, interviewees across primary and specialist care expressed emotional fatigue, especially regarding the failure of health and other government systems to improve housing and environmental conditions. Increased investment in these upstream causes has well‐understood implications for the primordial prevention of RHD and other infectious and chronic conditions [12]. These results demonstrate that this investment is also important to preserve the morale and motivation of healthcare providers who care for RHD patients.

Practical changes to support healthcare staff in WA could include improved interoperability between the WA register and health provider records systems; extension of existing processes for systematic rather than reactive information sharing between providers; and allocation of operational responsibility for ongoing RHD patient follow‐up within health services. Similar recommendations were made in the Australian RHD Guidelines and the RHD Endgame Strategy [11, 12]. These changes may alleviate the pressure that individual providers feel to keep track of patient information, especially in the context of a transient workforce and population.

### 4.1 Strengths and Limitations

This is the first mixed‐methods study to examine RHD‐related mortality in Australia. This approach facilitated a holistic understanding by situating the characteristics of the deceased group from the WA register within the context of health service delivery in WA. This research foregrounds the perspectives and experiences of WA RHD stakeholders, whose voices would have been unaccounted for if the study had relied on statistics alone. It is also the first study to examine RHD mortality in WA in detail, providing important insights for policymakers and public health professionals.

Information on the WA register is recorded manually by register staff and is dependent on clinician notification, which is known to be incomplete [31]. Data sourced from the register is therefore likely to underestimate both RHD cases and deaths. Australian RHD registers are more likely to detect cases among younger, Aboriginal, and northern Australian residents, who are likely overrepresented in the study population [30]. The WA Register was managed from the Kimberley region between 2009 and 2017, which has probably led to higher case ascertainment than for southern regions.

The interview sample size was limited by the small number of RHD stakeholders in WA. While saturation of themes was achieved by the final interview, it is possible that this analysis has missed alternative stakeholder perspectives on RHD care in WA.

## 5 Conclusion

This study combines quantitative and qualitative insights to suggest opportunities for preventing RHD‐related mortality in WA. Committed WA healthcare providers endeavour to overcome barriers and connect RHD patients with appropriate care. Trusting relationships between patients, families, and providers were identified as essential for this. The stability and motivation of this workforce require support to ensure sustainable access to best‐practice RHD care in WA. Necessary support includes resourcing and empowerment of the Aboriginal health workforce, cultural safety training for non‐Aboriginal staff, a shift in funding priorities towards primordial prevention, and practical changes to improve data sharing. These findings support recommendations raised in existing research and reports, including the RHD Endgame Strategy and the Australian RHD Guidelines [11, 12, 29]. Evidence generated by the present study highlights the need for national‐level recommendations to be implemented to reduce RHD mortality in WA.

## Author Contributions

**Virginia DeCourcy:** conceptualization, data curation, methodology, investigation, formal analysis, project administration, software, writing – original draft, visualization. **Daniel Hunt:** validation, visualization, writing – review and editing. **Ingrid Stacey:** conceptualization, formal analysis, methodology, software, validation, visualization, writing – review and editing. **Emma Haynes:** conceptualization, formal analysis, methodology, validation, writing – review and editing. **Beverley Paterson:** conceptualization, methodology, supervision, writing – review and editing. **Clare Huppatz:** writing – review and editing, resources, supervision. **Marisa Gilles:** conceptualization, funding acquisition, methodology, resources, supervision, validation, writing – review and editing. **Judith Katzenellenbogen:** conceptualization, funding acquisition, methodology, resources, supervision, validation, writing – review and editing.

## Disclosure

V.D.C. was a Master of Philosophy in Applied Epidemiology Scholar with the Australian National University and received a Master of Philosophy in Applied Epidemiology Scholarship funded by the WA Country Health Service. The Australian Bureau of Statistics data extract processing fee was jointly funded by the University of Western Australia and the WA Country Health Service.

## Ethics Statement

Ethics approval for this study was granted by both the WA Country Health Service Human Research Ethics Committee (RGS5578) and the WA Aboriginal Health Ethics Committee (HREC717) in August 2022.

## Conflicts of Interest

The authors declare no conflicts of interest.

## Supporting information


Data S1.

## Data Availability

The data that support the findings of this study are available on request from the corresponding author. The data are not publicly available due to privacy or ethical restrictions.

## References

[1] K. Kang , K. W. T. Chau , E. Howell , et al., “The Temporospatial Epidemiology of Rheumatic Heart Disease in Far North Queensland, Tropical Australia 1997–2017: Impact of Socioeconomic Status on Disease Burden, Severity and Access to Care,” PLoS Neglected Tropical Diseases 15, no. 1 (2021): e0008990. 33444355 10.1371/journal.pntd.0008990 PMC7840049

[2] R. Wyber , K. Noonan , C. Halkon , et al., “Ending Rheumatic Heart Disease in Australia: The Evidence for a New Approach,” Medical Journal of Australia 213, no. 10 (2020): S3–S31. 10.5694/mja2.50853 33190287

[3] S. Coffey , R. Roberts‐Thomson , A. Brown , et al., “Global Epidemiology of Valvular Heart Disease,” Nature Reviews Cardiology 18, no. 12 (2021): 853–864. 34172950 10.1038/s41569-021-00570-z

[4] I. Stacey , R. Seth , L. Nedkoff , et al., “Rheumatic Heart Disease Mortality in Indigenous and Non‐Indigenous Australians Between 2010 and 2017,” Heart 109, no. 13 (2023): 1025–1033. 36858807 10.1136/heartjnl-2022-322146

[5] S. M. Colquhoun , J. R. Condon , A. C. Steer , S. Q. Li , S. Guthridge , and J. R. Carapetis , “Disparity in Mortality From Rheumatic Heart Disease in Indigenous Australians,” Journal of the American Heart Association 4, no. 7 (2015): e001282, 10.1161/JAHA.114.001282. 26219562 PMC4608059

[6] J. Cannon , K. Roberts , C. Milne , and J. R. Carapetis , “Rheumatic Heart Disease Severity, Progression and Outcomes: A Multi‐State Model,” Journal of the American Heart Association 6, no. 3 (2017): e003498, 10.1161/JAHA.116.003498. 28255075 PMC5523987

[7] V. Y. F. He , J. R. Condon , A. P. Ralph , et al., “Long‐Term Outcomes From Acute Rheumatic Fever and Rheumatic Heart Disease: A Data‐Linkage and Survival Analysis Approach,” Circulation 134, no. 3 (2016): 222–232. 27407071 10.1161/CIRCULATIONAHA.115.020966 PMC4949009

[8] J. M. Katzenellenbogen , D. Bond‐Smith , R. J. Seth , et al., “Contemporary Incidence and Prevalence of Rheumatic Fever and Rheumatic Heart Disease in Australia Using Linked Data: The Case for Policy Change,” Journal of the American Heart Association 9, no. 19 (2020): e016851. 32924748 10.1161/JAHA.120.016851 PMC7792417

[9] E. Haynes , R. Walker , A. G. Mitchell , J. Katzenellenbogen , H. D'Antoine , and D. Bessarab , “Decolonizing Indigenous Health: Generating a Productive Dialogue to Eliminate Rheumatic Heart Disease in Australia,” Social Science & Medicine 277 (2021): 113829. 33895707 10.1016/j.socscimed.2021.113829

[10] W. Fogarty , M. Lovell , J. Langenberg , and M. Heron , Deficit Discourse and Strengths‐Based Approaches: Changing the Narrative of Aboriginal and Torres Strait Islander Health and Wellbeing (Lowitja Institute, 2018).

[11] RHD Australia , The 2020 Australian Guideline for Prevention, Diagnosis and Management of Acute Rheumatic Fever and Rheumatic Heart Disease (Menzies School of Health Research, 2020). 10.5694/mja2.50851 33190309

[12] END RHD Centre of Research Excellence , The RHD Endgame Strategy: The Blueprint to Eliminate Rheumatic Heart Disease in Australia by 2031 (Telethon Kids Institute, 2020).

[13] E. Haynes , M. Marawili , M. B. Marika , et al., “Living With Rheumatic Heart Disease at the Intersection of Biomedical and Aboriginal Worldviews,” International Journal of Environmental Research and Public Health 19, no. 8 (2022): 4650. 35457520 10.3390/ijerph19084650 PMC9025526

[14] E. Haynes , A. Mitchell , S. Enkel , R. Wyber , and D. Bessarab , “Voices Behind the Statistics: A Systematic Literature Review of the Lived Experience of Rheumatic Heart Disease,” International Journal of Environmental Research and Public Health 17, no. 4 (2020): 1347, 10.3390/ijerph17041347. 32093099 PMC7068492

[15] N. Wilson , Findings of Inquest Into the Deaths of RHD Doomadgee Cluster (Coroners Court of Queensland, 2023), https://www.courts.qld.gov.au/__data/assets/pdf_file/0006/770109/cif‐booth‐sandy‐george‐20230630.pdf.

[16] R. V. C. Fogliani , Record of Investigation Into Death of Mr. Yeeda (Coroner's Court of Western Australia, 2022), https://www.coronerscourt.wa.gov.au/_files/inquest_2022/Yeeda%20(Seth)%20finding.pdf.

[17] A. Mitchell , V. Wade , E. Haynes , J. Katzenellenbogen , and D. Bessarab , ““The World Is So White”: Improving Cultural Safety in Healthcare Systems for Australian Indigenous People With Rheumatic Heart Disease,” Australian and New Zealand Journal of Public Health 46, no. 5 (2022): 588–594. 35852387 10.1111/1753-6405.13219

[18] Western Australia Department of Health , WA Rheumatic Heart Disease Register (WA DoH, 2021), https://health.wa.gov.au/Articles/U_Z/WA‐rheumatic‐heart‐disease‐register.

[19] A. Hofer , S. Woodland , and C. Reeve , “Mortality due to Rheumatic Heart Disease in the Kimberley 2001–2010,” Australian and New Zealand Journal of Public Health 38, no. 2 (2014): 139–141. 24812716 10.1111/1753-6405.12112

[20] S. B. Davies , A. Hofer , and C. Reeve , “Mortality Attributable to Rheumatic Heart Disease in the Kimberley: A Data Linkage Approach,” Internal Medicine Journal 44, no. 11 (2014): 1074–1080. 25070793 10.1111/imj.12540

[21] Australian Institute of Health and Welfare , Acute Rheumatic Fever and Rheumatic Heart Disease in Australia 2017–2021 (AIHW, 2023).

[22] A. Durey and S. C. Thompson , “Reducing the Health Disparities of Indigenous Australians: Time to Change Focus,” BMC Health Services Research 12, no. 151 (2012): 1–11. 22682494 10.1186/1472-6963-12-151 PMC3431273

[23] J. Francis , H. Fairhurst , H. Hardefeldt , et al., “Echocardiographic Screening Detects Extremely High Prevalence of RHD in Australia,” Heart, Lung & Circulation 28 (2019): S51.

[24] I. Stacey , J. Hung , J. Cannon , et al., “Long‐Term Outcomes Following Rheumatic Heart Disease Diagnosis in Australia,” European Heart Journal Open 1, no. 3 (2021): oeab035. 35919882 10.1093/ehjopen/oeab035 PMC9242034

[25] D. Nolan‐Isles , R. Macniven , K. Hunter , et al., “Enablers and Barriers to Accessing Healthcare Services for Aboriginal People in New South Wales, Australia,” International Journal of Environmental Research and Public Health 18, no. 6 (2021): 3014. 33804104 10.3390/ijerph18063014 PMC7999419

[26] A. Anderson , B. Peat , J. Ryland , et al., “Mismatches Between Health Service Delivery and Community Expectations in the Provision of Secondary Prophylaxis for Rheumatic Fever in New Zealand,” Australian and New Zealand Journal of Public Health 43, no. 3 (2019): 294–299. 30908804 10.1111/1753-6405.12890

[27] E. Haynes , S. Noonan , A. Mitchell , et al., Investigating Factors That Impede and Enable Optimal Implementqtion of Regional Rheumatic Heart Disease Control in Primary Health Care Settings—Strategies and Interventions (University of Western Australia, 2022).

[28] C. Davy , A. Cass , J. Brady , et al., “Facilitating Engagement Through Strong Relationships Between Primary Healthcare and Aboriginal and Torres Strait Islander Peoples,” Australian and New Zealand Journal of Public Health 40, no. 6 (2016): 535–541. 27523395 10.1111/1753-6405.12553

[29] E. Haynes , J. M. Katzenellenbogen , S. Noonan , et al., “Is the Australian Primary Healthcare System Ready for the Rheumatic Heart Disease Endgame Strategy? Data Synthesis and Recommendations,” Australian and New Zealand Journal of Public Health 46, no. 5 (2022): 554–557. 35852386 10.1111/1753-6405.13259

[30] V. Kerrigan , S. Y. McGrath , R. M. Herdman , et al., “Evaluation of ‘Ask the Specialist’: A Cultural Education Podcast to Inspire Improved Healthcare for Aboriginal Peoples in Northern Australia,” Health Sociology Review 31, no. 2 (2022): 139–157. 35373706 10.1080/14461242.2022.2055484

[31] T. Agenson , J. M. Katzenellenbogen , R. Seth , et al., “Case Ascertainment on Australian Registers for Acute Rheumatic Fever and Rheumatic Heart Disease,” International Journal of Environmental Research and Public Health 17, no. 15 (2020): 1–23. 10.3390/ijerph17155505 PMC7432403 32751527

