# Supplementary material for: Connections Reduce Rheumatic Heart Disease‐Related Mortality in Western Australia: A Mixed Methods Study
Source: Aust J Rural Health. 2025 Mar 10;33(2):e70022. doi: 10.1111/ajr.70022 (PMC11891974; doi:10.1111/ajr.70022)
Supplement: Supplementary file 1 — Data S1. [file AJR-33-0-s001.zip › ajr70022-sup-0001-Supinfo/submission_Opportunities for Reducing RHD Mortality in Western Australia_supplements.docx]

## Supplement A: RHD-specific mortality rates in Western Australia, 2012-2021

#### Background

Research conducted by Australian colleagues demonstrates high RHD mortality rates among Aboriginal compared to non-Aboriginal people across five Australian jurisdictions(4). This analysis used similar methodology to focus on RHD mortality rates in Western Australia.

#### Methods

Age-specific and age-standardised RHD mortality rates were calculated for Western Australia across a ten-year period from 2012 to 2021, and compared across Aboriginal status, sex and time variables. State-wide RHD mortality rates have not been previously reported in WA.

ICD-coded cause of death data was provided by the Australian Bureau of Statistics (ABS) in April 2023. Deaths in people aged 64 or younger, where RHD was coded as the underlying cause or as an associated cause (ICD codes I05, I06, I07, I08, I09 and their subcodes) were included. Population denominators were sourced from publicly available ABS Estimated Resident Populations across the ten years. Age standardised mortality rates were calculated via direct age standardisation across three age strata using the WHO World Standard Population 2000-2025. 95% confidence intervals were generated for each rate and rate ratio.

In the cause of death data provided, the ABS randomly assigns cells with small values (other than zero count cells), in order to protect individual confidentiality. Therefore, some totals presented below have minor discrepancies across tables.

#### Results

Age-standardised mortality was 38 times higher among Aboriginal people compared to non-Aboriginal people across the ten years [Table S1]. Mortality was significantly higher among Aboriginal women than Aboriginal men, but there was no significant difference by sex for non-Aboriginal people [Table S2]. There was no significant difference in mortality between the two five-year periods for either Aboriginal or non-Aboriginal people [Table S3].

## Supplement B: Interview Guide

Note: The interviews will follow a semi-structured approach. While the below questions outline the proposed general structure of an interview, these may change and/or be omitted depending on the responses of an individual interviewee. Interviewees will not be discouraged from discussing topics, issues or subject matter outside of the below questions, and probing questions may be asked about these discussions, as outlined below.

***Can you tell me about your experience/work with RHD?***

Prompts if necessary:

- How long have you been working in this area?
- What different roles have you had?
- What work do you do as part of your current role?
- How do you engage with people with RHD?
- How does your experience relate to WA specifically? Are there differences in your experience/work in WA compared to other jurisdictions?

***Rheumatic Heart Disease is an umbrella term that captures a long journey that a person may go through, from a Strep A infection up to heart surgery in some cases, and can span many years. This journey can involve many different encounters with the health system and other associated institutions. In your experience, what parts of the health system do patients come into contact with and how does the system itself impact on their journey with RHD?***

Prompts if necessary:

- Who provides care to RHD patients?
- Are there multiple parts of the health system involved? Which other systems are involved with RHD patients?
- Do different parts of the health system work well together? How is the communication?
- What have you heard or observed from patients and communities about the way that the health system impacts on them?

***How have you seen the health system work effectively and/or contribute to positive patient outcomes?***

Prompts if necessary:

- Can you think of any programs or services that have made positive differences for patients?
- In your experience, which parts of the health system have worked well together?

***Where might the health system be going wrong?***

- In your experience, what kind of negative impacts do people with RHD experience as a result of interacting with the health system?
- When have you seen this happen and what factors were at play?

***The research area for this project is RHD mortality in particular. How might systemic factors be contributing to RHD-related mortality in WA?***

- *Build on answer to last question, to focus on mortality if relevant/appropriate

***As part of this research we’ve conducted some quantitative analyses using ABS data and information from the WA RHD Register. Those analyses suggest that ____________ . Is that consistent with your experience? How would you explain that relationship?***

- *Prompts will depend on preliminary quantitative findings

***What health system improvements do you think should be prioritised to help to avoid RHD-related mortality in the future?***

- What changes would you like to see in the parts of the health system you work in/with?
- Have you seen system changes work well elsewhere in Australia or overseas that you would like to see in WA?
- What changes/improvements do you think would have the biggest impact?
- What are the potential roadblocks to implementing these changes?

General prompts to encourage further discussion:

- Can you expand on that?
- When you said ________, what did you mean?
- Can you give me an example?
- Tell me more about [x issue].
- Why do you think [x issue] is the case?

After Interview Concludes

- The recording will be stopped when the interview comes to a natural conclusion.
- The interviewee will be thanked for their time and invited to get in touch with the research team if they think of other issues or themes they would like to add to the perspectives they have provided.
- The interviewee will be reminded to seek support if they have experienced any emotional distress as a result of the interview.
