# Supplementary material for: Connections Reduce Rheumatic Heart Disease‐Related Mortality in Western Australia: A Mixed Methods Study
Source: Aust J Rural Health. 2025 Mar 10;33(2):e70022. doi: 10.1111/ajr.70022 (PMC11891974; doi:10.1111/ajr.70022)
Supplement: Supplementary file 1 — Data S1. [file AJR-33-0-s001.zip › ajr70022-sup-0001-Supinfo/supp tables.docx]

#### Table S1. Age-specific and age-standardised Rheumatic heart disease deaths and death rate ratios (DRRs) in Western Australia 2012-2021, per 100,000 person-years, by Aboriginal status

|  | **Aboriginal** | | | **Non-Aboriginal** | | | **Rate Ratio** | |
| --- | --- | --- | --- | --- | --- | --- | --- | --- |
| **Age** | **n** | **Rate** | **95% CI** | **n** | **Rate** | **CI** | **DRR** | **95% CI** |
| 0 - 24 | 3 | 0.57 | 0.00 – 1.21 | 0 | 0.00 | - | - | - |
| 25 - 44 | 17 | 6.08 | 3.19 – 8.97 | 7 | 0.10 | 0.02 – 0.17 | 63.63 | 26.39 – 153.45 |
| 45 - 64 | 43 | 25.49 | 17.87 – 33.10 | 44 | 0.71 | 0.50 – 0.92 | 35.85 | 23.55 – 54.58 |
| Age-standardised rate | | | | | | | | |
| 0-64 | 63 | 14.87 | 10.93 – 18.80 | 51 | 0.39 | 0.28 – 0.49 | 38.38 | 26.53 – 55.52 |

#### Table S2. Age-specific and age-standardised Rheumatic heart disease deaths and death rate ratios (DRRs) in Western Australia 2012-2021, per 100,000 person-years, by Aboriginal status and sex

| **Sex** | **Female** | | | **Male** | | | **Rate Ratio** | |
| --- | --- | --- | --- | --- | --- | --- | --- | --- |
| **Age** | **n** | **Rate** | **CI** | **n** | **Rate** | **CI** | **DRR** | **CI** |
| Aboriginal | | | | | | | | |
| 0 - 24 | 1 | 0.39 | 0.00 – 1.14 | 2 | 0.74 | 0.00 – 1.77 | 0.52 | 0.05 – 5.72 |
| 25 - 44 | 10 | 7.31 | 2.78 – 11.84 | 7 | 4.91 | 1.27 – 8.54 | 1.49 | 0.57 – 3.91 |
| 45 - 64 | 31 | 34.82 | 22.56 – 47.07 | 12 | 15.06 | 6.54 – 23.58 | 2.31 | 1.19 – 4.50 |
| Non-Aboriginal | | | | | | | | |
| 0 - 24 | 0 | 0.00 | - | 0 | 0.00 | - | - | - |
| 25 - 44 | 4 | 0.11 | 0.00 – 0.22 | 1 | 0.03 | 0.00 – 0.08 | 4.10 | 0.46 – 36.69 |
| 45 - 64 | 18 | 0.58 | 0.31 – 0.85 | 26 | 0.84 | 0.52 – 1.16 | 0.69 | 0.38 – 1.26 |
| Age-standardised rate Aboriginal | | | | | | | | |
| 0-64 | 42 | 19.91 | 13.60 – 26.22 | 21 | 9.29 | 4.86 – 13.72 | 2.14 | 1.27 – 3.62 |
| Age-standardised rate Non-Aboriginal | | | | | | | | |
| 0-64 | 22 | 0.33 | 0.19 – 0.47 | 27 | 0.43 | 0.27 – 0.59 | 0.76 | 0.43 – 1.34 |

#### Table S3. Age-specific and age-standardised Rheumatic heart disease deaths and death rate ratios (DRRs) in Western Australia 2012-2021, per 100,000 person-years, by Aboriginal status and time period

| **Year** | **2012 - 2016** | | | **2017-2021** | | | **Rate Ratio** | |
| --- | --- | --- | --- | --- | --- | --- | --- | --- |
| **Age** | **n** | **Rate** | **CI** | **n** | **Rate** | **CI** | **DRR** | **CI** |
| Aboriginal | | | | | | | | |
| 0 - 24 | 2 | 0.77 | 0.00 – 1.85 | 2 | 0.74 | 0.00 – 1.76 | 1.04 | 0.15 – 7.44 |
| 25 - 44 | 1 | 0.75 | 0.00 – 2.23 | 13 | 8.86 | 4.04 – 13.67 | 0.09 | 0.01 – 0.65 |
| 45 - 64 | 19 | 24.34 | 13.40 – 35.29 | 24 | 26.47 | 15.88 – 37.06 | 0.92 | 0.50 – 1.68 |
| Non-Aboriginal | | | | | | | | |
| 0 - 24 | 0 | 0.00 | - | 0 | 0.00 | - | - | - |
| 25 - 44 | 6 | 0.17 | 0.03 – 0.30 | 3 | 0.08 | 0.00 – 0.17 | 2.05 | 0.51 – 8.18 |
| 45 - 64 | 19 | 0.64 | 0.35 – 0.92 | 25 | 0.78 | 0.47 – 1.09 | 0.82 | 0.45 – 1.48 |
| Age-standardised rate Indigenous | | | | | | | | |
| 0-64 | 22 | 12.55 | 7.05 – 18.05 | 39 | 16.31 | 10.77 – 21.85 | 1.30 | 0.77 – 2.19 |
| Age-standardised rate Non-Aboriginal | | | | | | | | |
| 0-64 | 25 | 0.37 | 0.22 – 0.52 | 28 | 0.42 | 0.26 – 0.57 | 1.18 | 0.65 – 1.92 |
